# Hybrid Closed-loop to Manage Gastroparesis in People With Type 1 Diabetes: a Case Series

**DOI:** 10.1177/19322968211035447

**Published:** 2021-08-11

**Authors:** Aideen Daly, Sara Hartnell, Charlotte K. Boughton, Mark Evans

**Affiliations:** 1 Wellcome Trust-MRC Institute of Metabolic Science, University of Cambridge, Cambridge, UK; 2 Wolfson Diabetes and Endocrine Clinic, Cambridge University Hospitals NHS Foundation Trust, Cambridge, UK

**Keywords:** continuous glucose monitoring, diabetes technology, gastroparesis, hybrid closed-loop, type 1 diabetes

## Abstract

**Background:**

Gastroparesis is associated with unpredictable gastric emptying and can lead to erratic glucose profiles and negative impacts on quality-of-life. Many people with gastroparesis are unable to meet glycemic targets and there is a need for new approaches for this population. Hybrid closed-loop systems improve glucose control and quality-of-life but evidence for their use in people with diabetic gastroparesis is limited.

**Methods:**

We present a narrative review of the challenges associated with type 1 diabetes management for people with gastroparesis and present a case series of 7 people with type 1 diabetes and gastroparesis. We compare glycemic control before and during the first 12 months of hybrid closed-loop therapy. Data were analyzed using electronic patient records and glucose management platforms. We also discuss future advancements for closed-loop systems that may benefit this population.

**Results:**

Five of 7 patients had data available for time in range before and during hybrid closed-loop therapy, and all had an improvement in percentage time in target glucose range, with the overall mean time in range increasing from 26.0% ± 15.7% to 58.4% ± 8.6% during HCL use, (*P* = .004). There were significant reductions in HbA1c (83 ± 9 mmol/mol to 71 ± 14 mmol/mol) and mean glucose from 13.0 ± 1.7 mmol/L (234 ± 31 mg/dL) to 10.0 ± 0.7 mmol/L (180 ± 13 mg/dL) with use of a hybrid closed-loop system. Importantly, this was achieved without an increase in time in hypoglycemia (*P* = .50).

**Conclusion:**

Hybrid closed-loop systems may represent a valuable approach to improve glycemic control for people with type 1 diabetes and gastroparesis. Prospective studies are required to confirm these findings.

## Introduction

Gastroparesis is a form of autonomic neuropathy resulting in delayed gastric emptying in the absence of mechanical gastric outlet obstruction.^1^ It is estimated that between 20% and 50% of people with type 1 diabetes (T1D) will develop the complication,^2^ which is thought to occur as a result of immune dysregulation causing loss of gastric pacemaker cells, fibrosis in muscle layers and loss of enteric nerves in people with diabetes.^3,4^ Cardinal symptoms include early satiety, nausea, vomiting and bloating^5^ and the associated burden of these symptoms has been shown to have a profound impact on quality of life and healthcare utilization by affected individuals.^1,6^ Diagnosis is established by demonstration of delayed gastric emptying and the absence of gastric outlet obstruction using scintigraphy, capsule endoscopy or isotope breath tests in symptomatic individuals. The results of these investigations may be confounded by medications and severe hyperglycemia, making interpretation challenging.^7^ Although it remains unclear whether optimization of glycemia can improve or reverse the symptoms or severity of gastroparesis, strategies to improve glycemic control remain a key management approach, alongside prokinetics, neuromodulators, dietary modification and antiemetics.^8,9^ Unfortunately, in many cases, symptoms are refractory to therapies leading to poor nutritional status and high levels of distress.^10^ For people with severe symptoms of gastroparesis, more invasive surgical procedures such as botulinum toxin injections or gastric electrical stimulation may be indicated, however evidence for these therapies is limited.^2,11^

Glucose management is particularly challenging for people with T1D and gastroparesis; people with gastroparesis have a higher risk of severe hypoglycemia and increased difficulty achieving recommended glycemic targets compared to people without gastroparesis.^12^ Delayed gastric emptying leads to unpredictable variability in prandial glucose excursions and can predispose to hyperglycemia hours after meal intake.^13^ Acute and chronic hyperglycemia have been shown to delay gastric emptying,^9^ highlighting a complex bidirectional relationship between glucose and gastric emptying. Optimization and timing of insulin dosing is difficult and there is a clear need to develop tools to enable people with gastroparesis to manage this more effectively.

There is mounting evidence that glucose responsive insulin delivery via hybrid closed-loop (HCL) systems results in improved glycemic control, greater quality of life and reduced diabetes burden for people with T1D.^14-17^ HCL systems require users to bolus manually for meals. A number of HCL systems have been approved for use by people with T1D, including Medtronic 670G/780G, Tandem t:slim X2 with Control IQ and CamAPS FX.^17^ The ability of these systems to manage high glycemic variability without increasing the risk of hypoglycemic events renders this an attractive option to simplify glucose management in people with gastroparesis.^18^

Evidence for the use of HCL systems by people with diabetic gastroparesis is limited and it is unclear whether HCL control algorithms can cope with the unpredictable arrival of glucose from meals into the bloodstream. In this report, we discuss the complexities of glycemic management for people with T1D and gastroparesis and present a case series evaluating use of HCL insulin delivery in this population. All patients were previously on insulin pump therapy and transitioned to the use of a HCL system to manage their diabetes.

## Methods

Electronic patient records and data from glucose management platforms (Medtronic Carelink and Diasend) were collected and analyzed for 7 patients with type 1 diabetes and gastroparesis from a single tertiary diabetes clinic and commenced on HCL therapy between January 2019 and October 2020.

Percentage of time in target range from 3.9 to 10.0 mmol/L (70-180 mg/dL), time with glucose <3.9 mmol/L (70 mg/dL) and <3.0 mmol/L (54 mg/dL), and time in hyperglycemia >10 mmol/L (180 mg/dL) whilst on insulin pump therapy ± CGM was assessed using available data from the 3 months prior to commencing HCL therapy. Mean glucose, measures of glucose variability and insulin dose were recorded if available on the glucose management platforms. For each patient, the same glucose metrics were analyzed using available data for the first 12 months of closed-loop therapy.

As this is a retrospective analysis, HbA1c values were not systematically collected to assess efficacy of the HCL system; the most recent HbA1c value prior to starting the HCL system was compared to the latest HbA1c available while using HCL. Data on other relevant medical history and weight were obtained from electronic health records.

All patients provided verbal consent for the use of their anonymized data in this report.

Glycemic data from before and after HCL therapy were compared using student *t*-test for normally distributed data and Wilcoxon matched-pairs signed rank test for non-normally distributed data; statistical analyses were performed using Prism 9, version 9.0.2 (134) (GraphPad Software, LLC). Data are reported as mean ± SD or median (IQR) and *P*-values of <.05 were considered statistically significant.

## Results

Seven adults with T1D and gastroparesis started HCL therapy between January 2019 and October 2020. Six patients used the Medtronic MiniMed 670G and one patient used CamAPS FX. All patients were still using the closed-loop system at the time of analysis (May 2021). Patient characteristics are summarized in Table 1.

**Table 1.** Patient Demographics.

Age (years)	46.0 (42.0, 46.5)
Gender	6 female, 1 male
Diabetes duration (years)	28.6 ± 11.4
Gastroparesis duration (years)	8.4 ± 4.8
Previous treatments for gastroparesis (no. of patients)	Botulinum toxin (3), Metoclopramide (2), Omeprazole (2), Ondansetron (1), Prochlorperazine (1), Gastric pacemaker (1), Pyloroplasty (1), Erythromycin (1), Loperamide (1), Buscopan (1), Domperidone (1), Percutaneous endoscopic jejunostomy (1)
Co-morbidities, *n* (%) of patients	
Retinopathy	7 (100)
Neuropathy	5 (71)
Peripheral vascular disease	2 (29)
Nephropathy	2 (29)
Cerebrovascular accident	2 (29)
Angina	1 (14)

Six patients were female and one was male. Median age was 46.0 (42.0, 46.5) years and mean duration of type 1 diabetes and gastroparesis was 28.6 ± 11.4 and 8.4 ± 4.8 years, respectively. All 7 patients had a clinically established diagnosis of gastroparesis refractory to optimized therapies including intragastric botulinum injections and pyloroplasty in one individual and insertion of a gastric pacemaker in another. Diagnosis of gastroparesis was confirmed using scintigraphy in 3 individuals and small bowel manometry in one. Information on diagnostic methods for 3 individuals was not available. Other micro- and macrovascular complications were common, including laser treated retinopathy (100%), nephropathy (29%), foot ulceration (29%), cerebrovascular accident (29%) and angina (14%). At the time of analysis, mean time using the HCL system was 671 ± 171 days.

### Glucose Metrics

**Figure 1.**
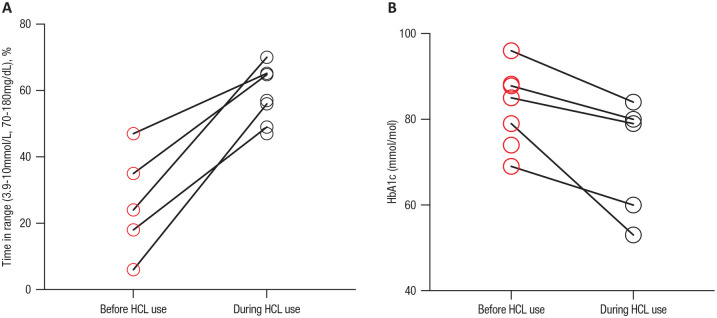
(A) Comparison of time in target glucose range for patients with gastroparesis before and during hybrid closed-loop therapy. (B) HbA1c values for patients with gastroparesis before and during hybrid closed-loop therapy. *Only 5 patients had available data for both pre-HCL and during HCL therapy.

**Figure 2.**
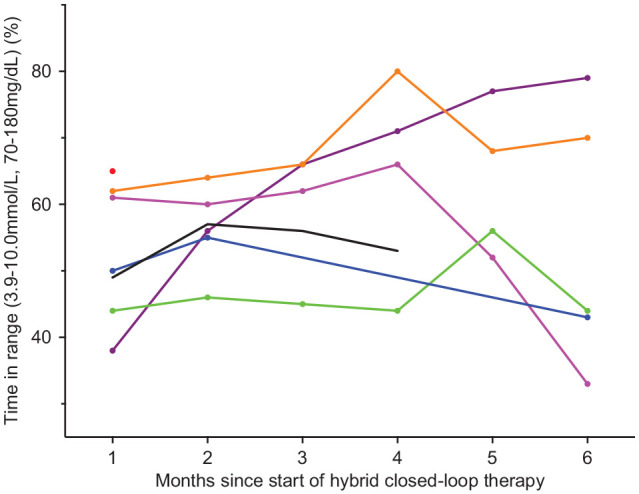
Time in range over the first 6 months of hybrid closed-loop therapy.

Data for time spent in target range (Figure 1A), hypoglycemia and hyperglycemia were available for 5 of 7 patients prior to starting closed-loop therapy. HbA1c values were available for all 7 patients prior to starting HCL therapy but only 5 had a HbA1c measured during HCL system use (see Figure 1B). For those with available data, mean percentage time in target glucose range between 3.9 and 10.0 mmol/l (70-180 mg/dL) prior to HCL therapy was 26.0 ± 15.7% which improved to 58.4% ± 8.6% during HCL use, (*P* = .004) (see Figures 1A and 2 for individual data). Time spent with glucose <3.9 mmol/L (70 mg/dL) and <3.0 mmol/L (54 mg/dL) was low both before and during HCL system use, with no significant increase in time spent in hypoglycemia with the use of the HCL system (Table 2). Mean percentage time spent in hyperglycemia (>10.0 mmol/L;180 mg/dL) was significantly lower during HCL therapy, with a reduction from 72.6% ± 14.3% before, to 40.1% ± 8.0% whilst using the HCL system (*P* = .003). Mean sensor glucose was lower during HCL therapy, 10.0 ± 0.7 mmol/L (180 ± 13 mg/dL) compared to 13.0 ± 1.7 mmol/L (234 ± 31 mg/dL) before (*P* = .005) and there were significant reductions in HbA1c from 83 + 9 mmol/mol (9.7% ± 3.0%) to 71 + 14 mmol/mol (8.6% ± 3.4%) during HCL therapy (see Figure 1B). Glucose metrics are summarized in Table 2.

**Table 2.** Glucose and Insulin Metrics Before and During Hybrid Closed-Loop Therapy.

	During insulin pump therapy ± CGM	During closed-loop therapy	*P*-Value
HbA1c, mmol/mol (%) (*n* = 7 pre-CL, *n* = 5 during CL)	83 ± 9 (9.7 ± 3.0)	71 ± 14 (8.6 ± 3.4)	.03
CGM metrics (*n* = 5 pre-HCL, *n* = 7 during HCL)			
Time between 3.9-10.0 mmol/L (70-180 mg/dL), %	26.0 ± 15.7	58.4 ± 8.6	.004
Time between 3.0-3.9 mmol/L (54-70 mg/dL), %	0.0 (0.0, 0.0)	1.0 (0.5, 1.0)	.5
Time <3.0 mmol/L (54 mg/dL), %	0.0 (0.0, 1.0)	0.0 (0.0, 0.0)	.5
Time >10.0 mmol/L (180 mg/dL), %	72.6 ± 14.3	40.1 ± 8.0	.003
Mean glucose, mmol/L (mg/dL)	13.0 ± 1.7 (234 ± 31)	10.0 ± 0.7 (180 ± 13)	.005
SD of glucose, mmol/L (mg/dL)	4.1 ± 0.6 (74 ± 11)	3.5 ± 0.7 (63 ± 13)	.02
CV of glucose, %	Not measured	36.8 ± 1.9	—
Insulin metrics (*n* = 7 pre-HCL and during HCL)			
Total daily insulin dose, units/day	39.7 ± 26.2	42.4 ± 27.6	.51
Total basal insulin dose, units/day	20.1 ± 14.7	24.8 ± 16.9	.16
Total bolus insulin dose, units/day	19.6 ± 14.2	17.1 ± 11.0	.64
Weight, kg	72.3 ± 14.0	72.2 ± 14.1	.36
Insulin requirement, units/kg/day	0.5 ± 0.3	0.6 ± 0.3	.51
No of days data	6.6 ± 10.6	173 ± 116.5	Not measured
Auto-mode use, %	—	66.4 ± 17.6	—

## Discussion

In an observational 30-year follow up from the Diabetes Control and Complications Trial (DCCT), the Epidemiology of Diabetes Interventions and Complications (EDIC) study demonstrated that individuals with T1D who had greater dysglycemia exposure (higher baseline HbA1c and longer duration of diabetes) were more likely to develop delayed gastric emptying.^19^ For people with established gastroparesis, however, data is conflicting on whether intensive glycemic control can modify the disease course.^8,20^ In a 12-year longitudinal study of 16 patients with T1D and 4 patients with type 2 diabetes (T2D), no differences were shown in gastric emptying or gastrointestinal symptoms despite lower mean glucose and lower HbA1c after a 12 year period.^21^ Similarly, Bharucha et al.^20^ investigated the use of overnight insulin infusions and 6 months of intensive insulin therapy in 30 patients with poorly controlled T2D, which resulted in a HbA1c reduction from 10.6% ± 0.3% to 9.0% ± 0.4%, but no effect on gastric emptying. To the contrary, another study by Laway et al. involving 30 women with newly diagnosed T2D and twenty age, weight and gender matched controls showed delayed gastric emptying in 90% of the former, and none of the control group. On optimization of glucose control, gastric emptying was shown to normalize in this study.^8^ Indeed, it has been proposed that diabetic gastroparesis may be over-diagnosed due to the fact that delayed gastric emptying can be observed in subjects without diabetes during induced hyperglycemia, suggesting this is a physiological response to higher glucose levels.^22^ Nevertheless, as the incidence of retinopathy, neuropathy and nephropathy is higher in those with gastroparesis compared to those without,^23^ optimization of glycemic control remains a priority for this group.

Prandial glucose excursions are a limiting factor for optimizing glucose control for many people with diabetes and they are affected by many variables including composition and macronutrient content of food, and gastric emptying, with the latter accounting for 35% of the variance in glucose levels.^13^ Delayed gastric emptying and mismatch between insulin action and food absorption can predispose to hypoglycemia, therefore intentional underestimation, delayed administration or omission of insulin doses is common, resulting in suboptimal long term glycemic control.^1,24,25^ Acute and chronic hyperglycemia has been shown to reduce antral motility and increase proximal gastric adherence, causing delayed gastric emptying and perpetuating the cycle.^9,26^ These challenges have been recognized and there is wide acknowledgement of the need for novel treatment approaches to manage the high glucose variability seen in this group.

Strategies to improve post-prandial glucose excursions for people with diabetes include administration of insulin boluses approximately 15-20 minutes in advance of the meal.^27^ This approach is often unsuitable for individuals with gastroparesis due to unpredictable food intake and variable gastric emptying predisposing to early hypoglycemia. Ingestion of regular small meals with concomitant administration of rapid acting insulin and frequent glucose monitoring is a commonly adopted approach, however this poses a significant management burden for affected individuals and can be difficult to maintain.^2^ Addition of insulin for fat and protein content, and for those on insulin pump therapy, dual-wave boluses or frequent small boluses to match glycemic excursions^28^ are strategies that have been shown to effectively reduce post-prandial glucose excursions but are associated with high rates of hypoglycemia.^29,30^

Advances in diabetes technologies over recent years, including continuous glucose monitoring (CGM) devices and continuous subcutaneous insulin infusions (CSII) has enabled closer observation of glucose trends and allowed for greater flexibility in insulin delivery patterns to match glucose absorption.^25^ CGM alarms for out-of-range glucose values and arrows to indicate trends in glucose values increase confidence to intensify insulin therapy without the fear of hypoglycemia and allow for prospective insulin titration in an attempt to minimise glycemic excursions.^2^ For people with suspected gastroparesis, analysis of CGM data has also been postulated as a useful tool to aid diagnosis.^31^

Studies evaluating the use of insulin pump therapy with or without CGM in people with type 1 and type 2 diabetes and gastroparesis have shown significant improvements in glycemic control, reduced glycemic variability and lower healthcare resource use compared to those on multiple daily injections (MDI).^25,32^ Although the National Institute for Health and Care Excellence (NICE) does not provide specific guidance on insulin pump therapy for people with gastroparesis, guidelines state that people with T1D and disabling hypoglycemia or HbA1c above target despite a high level of care can be considered for insulin pump therapy, which applies to many individuals with the condition.

Despite an increasing body of evidence that HCL systems reduce the burden of diabetes management and improve glycemic outcomes for people with T1D, little is known about the efficacy and safety of HCL system use in people with diabetic gastroparesis. Data presented in this case series suggest that established benefits of HCL systems in people with T1D can also be seen in people with diabetic gastroparesis, despite unpredictable food absorption and higher glucose variability. With the increased complexity of diabetes management and symptomatic burden, people with gastroparesis may represent a group that could reap particular benefit from HCL system use. Across the 7 users of the HCL system in this case series, and with an average of 671 days use, all patients remained on the closed-loop system at the time of analysis, suggesting a high degree of treatment satisfaction.

To our knowledge, only one previous study reports on the use of HCL systems in people with T1D and gastroparesis. Kaur et al. reported the use of the Medtronic MiniMed 670G HCL system in 5 adults with T1D and gastroparesis and compared outcomes with 9 age, sex, and diabetes duration matched adults with T1D but without gastroparesis. Following 6 months of use, improvements in time in range and HbA1c reduction were comparable between those with and without gastroparesis.^33^ It is worth noting, however, that average time spent in target glucose range at baseline as reported by Kaur et al. was much higher than we observed in this case series at 55.4% (IQR 43.7, 60.7) vs 26% ± 15.7%, respectively, Furthermore, duration of gastroparesis in this case series was almost double that of the patients reported by Kaur et al., suggesting a more challenging group to treat reflected in this case series. Larger prospective randomized controlled trials are required to further investigate the efficacy, safety and cost-effectiveness of HCL systems in this group.

### Advancements of HCL Systems Which Could Benefit People With Gastroparesis

Advances in technology and bioengineering have enabled more precise matching of insulin requirements to glucose excursions, which may be of particular benefit for people with gastroparesis.

### Ultra-Rapid-Acting Insulin Analogues

Faster acting insulins, with addition of excipients to promote a faster on and faster off profile have become available over recent years, including faster insulin Aspart (Fiasp) and ultra-rapid Lispro (Lyumjev).^34,35^ Both analogues show accelerated absorption after subcutaneous administration compared to standard insulin and for Fiasp, earlier onset, doubling of initial exposure and an up to 2.5 fold increase in glucose lowering effect.^36^ To our knowledge, no studies have assessed the potential benefits of faster-acting insulin compared to standard insulin in people with gastroparesis and further studies in this population are warranted.

### Slowly Absorbed Meal (CamAPS FX)

Macronutrient content of food is a key factor affecting gastric emptying and foods with a high fat or protein load are known to delay gastric emptying by causing the release of peptides such as glucagon like peptide-1 (GLP-1), gastric inhibitory polypeptide (GIP) and cholecystokinin (CCK).^37^ Resultant delayed hyperglycemia and insulin resistance^38^ leads to glucose patterns similar to those typically observed in people with gastroparesis. The addition of a ‘slowly absorbed meal’ function on the CamAPS FX^39^ HCL system has been designed to match delayed and prolonged glucose excursions more closely with insulin delivery directed over a 3 to 4 hour period in response to rising glucose, but also maintains the ability to reduce or suspend insulin delivery if glucose is falling. This feature might be particularly useful for people with gastroparesis who have unpredictable absorption following meal intake.

### Closed-Loop Systems Which Allow Extended Bolus

Tandem t:slim X2 with Control-IQ Technology is the only commercially available hybrid closed-loop system which has the capacity to deliver an extended bolus,^40^ which allows for insulin delivery over a 2-hour period for slowly absorbed carbohydrates, and is particularly beneficial for those with gastroparesis. Future systems which enable extended or dual wave boluses to exist within a closed-loop system, and to deliver the bolus over a longer time period may further improve performance.

### Dual Hormone Artificial Pancreas Systems

To further advance the demonstrated benefits of hybrid closed-loop systems for people with T1D, there has been growing interest in dual-hormone systems which enable co-administration of insulin with other hormones and analogues such as glucagon, pramlintide or liraglutide.^41^ Randomized controlled trials have shown improved glucose control and lower incidence of hypoglycemia with an insulin and glucagon dual hormone system compared to single hormone systems, suggesting particular benefit for people with frequent hypoglycemia.^42^ There are several factors limiting widespread adoption of dual hormone systems, including the instability of glucagon in liquid form necessitating 24-hourly glucagon reservoir refills, the requirement to wear 2 pumps or reservoirs, and gastro-intestinal side effects of the hormone. Adjunctive pramlintide and GLP-1 both reduce post-prandial excursions and improve glucose control by slowing carbohydrate appearance by delaying gastric emptying, which may limit their use in people with gastroparesis. For people with gastroparesis, side effects of glucagon and pramlintide which include nausea, vomiting and early satiety may be particularly troublesome.^43,44^

### Future Directions

Although HCL systems have undoubtedly reduced the burden of diabetes management for people with T1D, the requirement for meal announcement for carbohydrate intake remains a limiting factor. Unpredictable meal absorption for people with gastroparesis adds a further layer of complexity, with uncertainty surrounding optimal bolus timing and fear of hypoglycemia if there is a mismatch between insulin administered and glucose excursions. Removing the requirement to manually enter carbohydrates at mealtimes allows the system to work as a ‘fully closed-loop’ system. Studies investigating the use of fully closed-loop systems in T1D have shown this to be a safe and effective approach to achieving target glucose outside post-prandial periods, however mitigation of post-prandial hyperglycemia remains a challenge.^45-47^ Further studies are ongoing (NCT04877730, NCT04545567) to investigate fully closed-loop, and with the advent of faster acting insulins and dual-hormone systems, fully closed-loop therapy for T1D may become a reality in the near future.

### Limitations

In our small single center case-series all but one of the patients were female, which limits the generalizability of our findings. A female gender bias is acknowledged for diabetic gastroparesis in the literature.^48^ The patients in this case series used 2 different HCL systems reflecting real-world patient preference.

Different investigations were used to diagnose gastroparesis among the 7 patients in this case series. While this reflects the heterogeneity in the diagnostic approach to this condition, it also limits the ability to reliably confirm the diagnosis in all cases in this cohort.

Due to the retrospective approach, glycemic metrics including HbA1c and time in range were evaluated at variable timepoints prior to, and after starting HCL for each patient. Furthermore, no assessment of gastroparesis symptomatology or qualitative assessments on user perceptions of the HCL system were undertaken, therefore we are unable to conclude if gastroparesis symptoms or the burden of diabetes management was reduced in this group.

This study is hypothesis generating and underpowered to draw any reliable conclusions on the usefulness of HCL systems in this group. Prospective randomized studies are required to evaluate this further.

## Conclusions

Achieving glycemic targets with conventional insulin therapies is challenging for people with diabetes, and for people with gastroparesis there are additional complicating factors. The development of hybrid closed-loop systems has reduced the burden of diabetes management for people with T1D by enabling glucose responsive insulin delivery, resulting in improved glycemic control and greater quality of life for users. Prospective randomized studies are required to establish if this is an effective and user-friendly option for people with T1D and gastroparesis.
